# 1-Ethyl-3-methyl-1*H*-imidazol-3-ium spiro­penta­borate

**DOI:** 10.1107/S1600536813034363

**Published:** 2014-01-18

**Authors:** T. Gannon Parker, Divya Pubbi, Austin Beehler, Thomas E. Albrecht-Schmitt

**Affiliations:** a Department of Chemistry and Biochemistry, Florida State University, 95 Chieftain Way, Tallahassee, Florida 32306, USA

## Abstract

In the anion of the title compound, (C_6_H_11_N_2_)[B_5_O_6_(OH)_4_], both six-membered borate rings adopt a flattened boat conformation with the spiro-B atom and its opposite O atom deviating from the remainders of the rings by 0.261 (3)/0.101 (2) and 0.160 (3)/0.109 (2) Å, respectively. The imidazolium cation also deviates from planarity due to rotation of the ethyl group (as indicated by the C—N—C—C torsion angle) by 71.4 (2)° out of the plane of the heterocycle. In the crystal, the anions are connected in a three-dimensional network through O—H⋯O hydrogen bonds, forming channels along the *a-*axis direction. The cations are situated in the channels, forming C—H⋯O hydrogen bonds with the anions.

## Related literature   

Several compounds with the same anion as the title compound, in addition to several anions that bear some similarities to it, have been prepared previously with a number of different cations. For an extensive analysis of these oxoboron compounds, please refer to the review by Lin & Yang (2011 ▶). The ionic liquid 1-ethyl-3-methyl­imidazolium bromide, from which the cation of the title compound originates, is one of the most common and easily synthesized ionic liquids available. For an extensive review of ionic liquid chemistry, including details on the preparation of imidazolium-based ionic liquids, please refer to the text by Wasserscheid & Welton (2003 ▶).
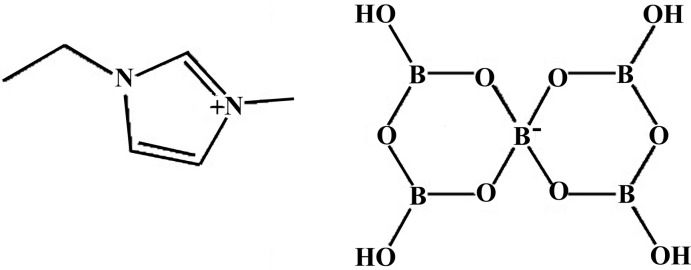


## Experimental   

### 

#### Crystal data  

C_6_H_11_N_2_^+^·B_5_H_4_O_10_^−^
*M* *_r_* = 329.25
Monoclinic, 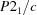

*a* = 9.3599 (12) Å
*b* = 15.1128 (19) Å
*c* = 10.4770 (13) Å
β = 92.181 (2)°
*V* = 1480.9 (3) Å^3^
*Z* = 4
Mo *K*α radiation
μ = 0.13 mm^−1^
*T* = 100 K
0.3 × 0.2 × 0.1 mm

#### Data collection  

Bruker D8 Quest diffractometer
Absorption correction: multi-scan (*SADABS*; Sheldrick, 1997 ▶) *T*_min_ = 0.970, *T*_max_ = 0.987
22780 measured reflections
3404 independent reflections
2902 reflections with *I* > 2σ(*I*)
*R*_int_ = 0.034

#### Refinement  

*R*[*F* ^2^ > 2σ(*F* ^2^)] = 0.033
*wR*(*F* ^2^) = 0.088
*S* = 1.04
3404 reflections
226 parameters
H atoms treated by a mixture of independent and constrained refinement
Δρ_max_ = 0.27 e Å^−3^
Δρ_min_ = −0.22 e Å^−3^

### 

Data collection: *SMART* (Bruker, 2000 ▶); cell refinement: *SAINT*; data reduction: *SAINT* and *XPREP* (Bruker, 1999 ▶); program(s) used to solve structure: *SHELXS97* (Sheldrick, 2008 ▶); program(s) used to refine structure: *SHELXL97* (Sheldrick, 2008 ▶); molecular graphics: *SHELXP97* (2008 ▶) and *CrystalMaker* (Kohn, 1995 ▶); software used to prepare material for publication: *publCIF* (Westrip, 2010 ▶).

## Supplementary Material

Crystal structure: contains datablock(s) I. DOI: 10.1107/S1600536813034363/ld2114sup1.cif

Structure factors: contains datablock(s) I. DOI: 10.1107/S1600536813034363/ld2114Isup2.hkl

CCDC reference:

Additional supporting information:  crystallographic information; 3D view; checkCIF report

## Figures and Tables

**Table 1:** Hydrogen-bond geometry (Å, °)

*D*—H⋯*A*	*D*—H	H⋯*A*	*D*⋯*A*	*D*—H⋯*A*
O7—H1⋯O9^i^	0.88 (2)	1.84 (2)	2.7169 (12)	172.8 (17)
O8—H2⋯O2^ii^	0.861 (18)	1.843 (18)	2.7022 (12)	174.9 (17)
O9—H3⋯O3^iii^	0.87 (2)	1.80 (2)	2.6687 (11)	174.3 (18)
O10—H4⋯O4^iv^	0.85 (2)	1.90 (2)	2.7426 (12)	173.9 (19)
C1—H1*A*⋯O5^iii^	0.95	2.14	3.0265 (14)	155
C4—H4*C*⋯O10^ii^	0.98	2.47	3.4456 (16)	175

Symmetry codes: (i) 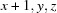; (ii) 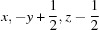; (iii) 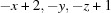; (iv) 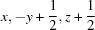.

**Fig. 1.**
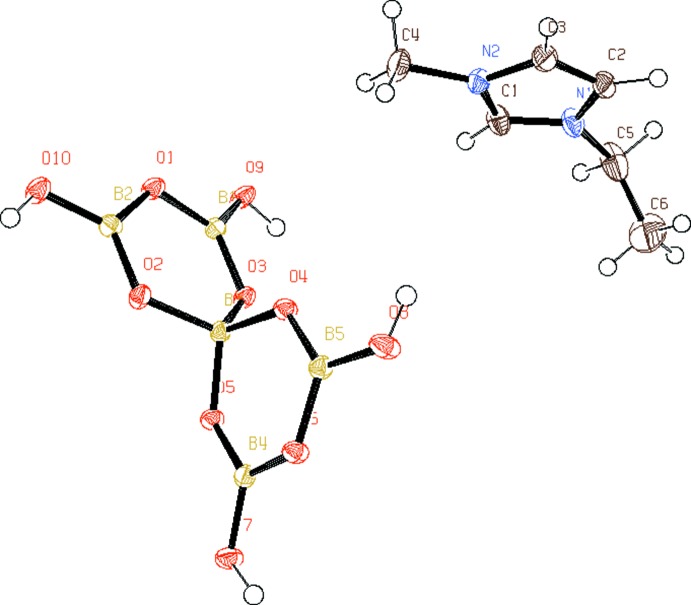
The molecular structure of 1-ethyl-3-methylimidazolium spiropentaborate including atomic numbering and 50% probability ellipsoids.

**Fig. 2.**
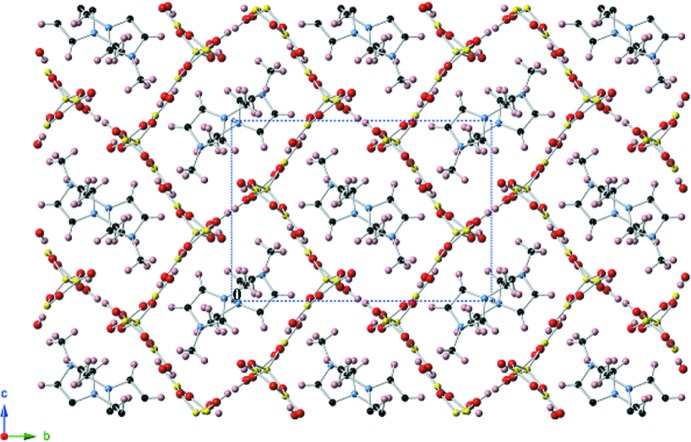
The view normal to the bc-plane showing the channels along which the cations reside. Hydrogen atoms not participating in hydrogen bonding have been omitted for clarity.

## supplementary crystallographic information

### 2. Experimental

The title compound formed as a byproduct in the ionothermal flux synthesis of a lanthanide borate cluster. 50.2 mg of erbium (III) oxide (Atomergic Chemetals Corp., 99.9 %), 97.2 mg of boric acid (EMD, 99.5 %) and 766 mg of 1-ethyl-3-methyl­imidazolium bromide were loaded into a PTFE-lined stainless steel autoclave with an inter­nal volume of 23 mL in the presence of 100 mL of water for counterpressure. After heating for 3 days at 150 °C, the autoclave was cooled to 25 °C at a rate of 5 °C per hour. Colorless blocks of the title compound suitable for single crystal X-ray diffraction were isolated from the reaction.

### 2.2. Refinement

Crystal data, data collection and structure refinement details are summarized in Table 1.

### 4. Refinement

H atoms of hydroxy groups were found in a difference Fourier synthesis and refined isotropically. The rest of the H atoms were calculated geometrically and refined within a riding/rotating model with *U*_iso_ = 1.2*U*_iso/eq_ of the adjacent carbon atom (1.5 for CH_3_ group).

### Crystal data

**Table:**

C_6_H_11_N_2_^+^·B_5_H_4_O_10_^−^	*F*(000) = 680
*M**_r_* = 329.25	*D*_x_ = 1.477 Mg m^−^^3^
Monoclinic, *P*2_1_/*c*	Mo *K*α radiation, λ = 0.71073 Å
Hall symbol: -P 2ybc	Cell parameters from 3404 reflections
*a* = 9.3599 (12) Å	θ = 2.2–27.5°
*b* = 15.1128 (19) Å	µ = 0.13 mm^−^^1^
*c* = 10.4770 (13) Å	*T* = 100 K
β = 92.181 (2)°	Block, colorless
*V* = 1480.9 (3) Å^3^	0.3 × 0.2 × 0.1 mm
*Z* = 4	

### Data collection

**Table:**

Bruker D8 Quest diffractometer	3404 independent reflections
Radiation source: Iµs microfocused	2902 reflections with *I* > 2σ(*I*)
Graphite monochromator	*R*_int_ = 0.034
0.5° wide ω exposures scans	θ_max_ = 27.5°, θ_min_ = 2.2°
Absorption correction: multi-scan (*SADABS*; Sheldrick, 1997)	*h* = −12→12
*T*_min_ = 0.970, *T*_max_ = 0.987	*k* = −19→19
22780 measured reflections	*l* = −13→13

### Refinement

**Table:**

Refinement on *F*^2^	Primary atom site location: structure-invariant direct methods
Least-squares matrix: full	Secondary atom site location: difference Fourier map
*R*[*F*^2^ > 2σ(*F*^2^)] = 0.033	Hydrogen site location: difference Fourier map
*wR*(*F*^2^) = 0.088	H atoms treated by a mixture of independent and constrained refinement
*S* = 1.04	*w* = 1/[σ^2^(*F*_o_^2^) + (0.0442*P*)^2^ + 0.5004*P*] where *P* = (*F*_o_^2^ + 2*F*_c_^2^)/3
3404 reflections	(Δ/σ)_max_ = 0.001
226 parameters	Δρ_max_ = 0.27 e Å^−^^3^
0 restraints	Δρ_min_ = −0.22 e Å^−^^3^

### Special details

**Table:**

Geometry. All e.s.d.'s (except the e.s.d. in the dihedral angle between two l.s. planes) are estimated using the full covariance matrix. The cell e.s.d.'s are taken into account individually in the estimation of e.s.d.'s in distances, angles and torsion angles; correlations between e.s.d.'s in cell parameters are only used when they are defined by crystal symmetry. An approximate (isotropic) treatment of cell e.s.d.'s is used for estimating e.s.d.'s involving l.s. planes.
Refinement. Refinement of *F*^2^ against ALL reflections. The weighted *R*-factor *wR* and goodness of fit *S* are based on *F*^2^, conventional *R*-factors *R* are based on *F*, with *F* set to zero for negative *F*^2^. The threshold expression of *F*^2^ > σ(*F*^2^) is used only for calculating *R*-factors(gt) *etc*. and is not relevant to the choice of reflections for refinement. *R*-factors based on *F*^2^ are statistically about twice as large as those based on *F*, and *R*- factors based on ALL data will be even larger.

### Fractional atomic coordinates and isotropic or equivalent isotropic displacement parameters (Å^2^)

**Table:**

	*x*	*y*	*z*	*U*_iso_*/*U*_eq_	
O1	0.92508 (8)	0.16731 (5)	0.69807 (7)	0.01589 (17)	
O6	1.49330 (8)	0.16170 (5)	0.52890 (7)	0.01567 (17)	
O3	1.08727 (8)	0.08183 (5)	0.57803 (7)	0.01407 (17)	
O5	1.33102 (8)	0.07833 (5)	0.65159 (7)	0.01398 (17)	
O2	1.17600 (8)	0.18727 (5)	0.73424 (7)	0.01490 (17)	
O4	1.24439 (8)	0.19660 (5)	0.51455 (7)	0.01469 (17)	
O9	0.83683 (8)	0.05749 (6)	0.56047 (8)	0.01843 (18)	
O7	1.57125 (9)	0.03768 (5)	0.65252 (8)	0.01789 (18)	
O8	1.40915 (9)	0.26406 (6)	0.37734 (8)	0.02090 (19)	
O10	1.00812 (9)	0.25571 (6)	0.86757 (8)	0.01941 (19)	
B1	0.95195 (13)	0.10211 (8)	0.60986 (11)	0.0140 (2)	
B3	1.21157 (12)	0.13593 (8)	0.61957 (11)	0.0128 (2)	
B2	1.03975 (13)	0.20424 (8)	0.76705 (12)	0.0146 (2)	
B4	1.46484 (13)	0.09275 (8)	0.61107 (11)	0.0136 (2)	
B5	1.37948 (13)	0.20780 (8)	0.47349 (12)	0.0146 (2)	
N2	0.69980 (10)	0.11782 (7)	0.14371 (9)	0.0194 (2)	
N1	0.70973 (12)	0.02735 (7)	−0.01465 (9)	0.0227 (2)	
C2	0.73105 (14)	0.11083 (8)	−0.06212 (11)	0.0217 (3)	
H2A	0.7474	0.1259	−0.1484	0.026*	
C3	0.72443 (13)	0.16723 (8)	0.03697 (11)	0.0222 (3)	
H3A	0.7349	0.2297	0.0336	0.027*	
C5	0.7149 (2)	−0.05582 (9)	−0.08757 (13)	0.0378 (4)	
H5A	0.6548	−0.0500	−0.1669	0.045*	
H5B	0.6752	−0.1044	−0.0363	0.045*	
C4	0.68454 (14)	0.15161 (9)	0.27388 (12)	0.0270 (3)	
H4A	0.6555	0.1033	0.3296	0.041*	
H4B	0.6117	0.1982	0.2730	0.041*	
H4C	0.7762	0.1759	0.3059	0.041*	
C1	0.69163 (14)	0.03353 (9)	0.11043 (11)	0.0240 (3)	
H1A	0.6755	−0.0146	0.1664	0.029*	
C6	0.8655 (3)	−0.07868 (12)	−0.12121 (18)	0.0576 (5)	
H6A	0.9059	−0.0300	−0.1701	0.086*	
H6B	0.8647	−0.1328	−0.1727	0.086*	
H6C	0.9238	−0.0881	−0.0427	0.086*	
H1	1.656 (2)	0.0489 (12)	0.6215 (16)	0.042 (5)*	
H2	1.3336 (19)	0.2818 (11)	0.3353 (16)	0.034 (4)*	
H3	0.864 (2)	0.0145 (13)	0.5119 (18)	0.047 (5)*	
H4	1.081 (2)	0.2741 (13)	0.9101 (19)	0.049 (5)*	

### Atomic displacement parameters (Å^2^)

**Table:**

	*U*^11^	*U*^22^	*U*^33^	*U*^12^	*U*^13^	*U*^23^
O1	0.0107 (4)	0.0196 (4)	0.0174 (4)	0.0003 (3)	0.0014 (3)	−0.0044 (3)
O6	0.0100 (4)	0.0192 (4)	0.0179 (4)	−0.0003 (3)	0.0018 (3)	0.0048 (3)
O3	0.0102 (4)	0.0169 (4)	0.0152 (4)	−0.0010 (3)	0.0019 (3)	−0.0035 (3)
O5	0.0107 (4)	0.0162 (4)	0.0152 (4)	0.0002 (3)	0.0019 (3)	0.0025 (3)
O2	0.0114 (4)	0.0191 (4)	0.0142 (4)	−0.0019 (3)	0.0014 (3)	−0.0035 (3)
O4	0.0113 (4)	0.0176 (4)	0.0153 (4)	0.0011 (3)	0.0016 (3)	0.0032 (3)
O9	0.0108 (4)	0.0223 (4)	0.0223 (4)	−0.0012 (3)	0.0028 (3)	−0.0094 (3)
O7	0.0114 (4)	0.0211 (4)	0.0213 (4)	0.0019 (3)	0.0028 (3)	0.0056 (3)
O8	0.0121 (4)	0.0272 (5)	0.0234 (4)	0.0007 (3)	0.0016 (3)	0.0115 (3)
O10	0.0124 (4)	0.0260 (4)	0.0199 (4)	−0.0009 (3)	0.0017 (3)	−0.0088 (3)
B1	0.0125 (5)	0.0162 (6)	0.0135 (5)	0.0004 (4)	0.0015 (4)	0.0005 (4)
B3	0.0106 (5)	0.0158 (6)	0.0121 (5)	−0.0008 (4)	0.0016 (4)	−0.0008 (4)
B2	0.0132 (6)	0.0156 (6)	0.0152 (6)	−0.0008 (4)	0.0011 (4)	0.0012 (4)
B4	0.0131 (5)	0.0154 (6)	0.0124 (5)	−0.0006 (4)	0.0011 (4)	−0.0008 (4)
B5	0.0132 (6)	0.0158 (6)	0.0148 (5)	−0.0007 (4)	0.0010 (4)	−0.0003 (4)
N2	0.0180 (5)	0.0238 (5)	0.0164 (5)	0.0005 (4)	0.0032 (4)	0.0010 (4)
N1	0.0336 (6)	0.0176 (5)	0.0170 (5)	−0.0006 (4)	0.0005 (4)	0.0030 (4)
C2	0.0284 (6)	0.0190 (6)	0.0181 (5)	0.0014 (5)	0.0042 (5)	0.0055 (4)
C3	0.0260 (6)	0.0193 (6)	0.0214 (6)	0.0011 (5)	0.0042 (5)	0.0037 (5)
C5	0.0731 (11)	0.0168 (6)	0.0230 (6)	−0.0010 (7)	−0.0052 (7)	0.0001 (5)
C4	0.0280 (7)	0.0351 (7)	0.0183 (6)	−0.0005 (5)	0.0064 (5)	−0.0040 (5)
C1	0.0309 (7)	0.0228 (6)	0.0184 (6)	−0.0039 (5)	0.0009 (5)	0.0048 (5)
C6	0.0941 (15)	0.0324 (9)	0.0470 (10)	0.0266 (9)	0.0111 (10)	−0.0080 (7)

### Geometric parameters (Å, º)

**Table:**

O1—B1	1.3805 (14)	N2—C1	1.3221 (17)
O1—B2	1.3881 (14)	N2—C3	1.3715 (15)
O6—B5	1.3822 (14)	N2—C4	1.4683 (15)
O6—B4	1.3839 (14)	N1—C1	1.3311 (15)
O3—B1	1.3571 (14)	N1—C2	1.3735 (15)
O3—B3	1.4740 (14)	N1—C5	1.4726 (16)
O5—B4	1.3553 (14)	C2—C3	1.3464 (17)
O5—B3	1.4463 (14)	C2—H2A	0.9500
O2—B2	1.3578 (14)	C3—H3A	0.9500
O2—B3	1.4788 (13)	C5—C6	1.506 (3)
O4—B5	1.3615 (14)	C5—H5A	0.9900
O4—B3	1.4736 (14)	C5—H5B	0.9900
O9—B1	1.3569 (14)	C4—H4A	0.9800
O9—H3	0.87 (2)	C4—H4B	0.9800
O7—B4	1.3566 (15)	C4—H4C	0.9800
O7—H1	0.88 (2)	C1—H1A	0.9500
O8—B5	1.3550 (15)	C6—H6A	0.9800
O8—H2	0.861 (18)	C6—H6B	0.9800
O10—B2	1.3512 (15)	C6—H6C	0.9800
O10—H4	0.85 (2)		
			
B1—O1—B2	118.59 (9)	C1—N1—C2	108.53 (10)
B5—O6—B4	118.51 (9)	C1—N1—C5	125.35 (11)
B1—O3—B3	122.39 (9)	C2—N1—C5	126.02 (11)
B4—O5—B3	123.09 (9)	C3—C2—N1	106.90 (10)
B2—O2—B3	123.19 (9)	C3—C2—H2A	126.6
B5—O4—B3	122.40 (9)	N1—C2—H2A	126.6
B1—O9—H3	110.2 (12)	C2—C3—N2	107.35 (11)
B4—O7—H1	114.9 (12)	C2—C3—H3A	126.3
B5—O8—H2	112.8 (11)	N2—C3—H3A	126.3
B2—O10—H4	113.9 (13)	N1—C5—C6	111.52 (14)
O9—B1—O3	121.94 (10)	N1—C5—H5A	109.3
O9—B1—O1	116.60 (10)	C6—C5—H5A	109.3
O3—B1—O1	121.41 (10)	N1—C5—H5B	109.3
O5—B3—O4	111.49 (9)	C6—C5—H5B	109.3
O5—B3—O3	109.22 (9)	H5A—C5—H5B	108.0
O4—B3—O3	108.02 (8)	N2—C4—H4A	109.5
O5—B3—O2	108.86 (9)	N2—C4—H4B	109.5
O4—B3—O2	109.87 (9)	H4A—C4—H4B	109.5
O3—B3—O2	109.35 (8)	N2—C4—H4C	109.5
O10—B2—O2	122.81 (10)	H4A—C4—H4C	109.5
O10—B2—O1	116.70 (10)	H4B—C4—H4C	109.5
O2—B2—O1	120.49 (10)	N2—C1—N1	108.60 (10)
O5—B4—O7	118.49 (10)	N2—C1—H1A	125.7
O5—B4—O6	121.20 (10)	N1—C1—H1A	125.7
O7—B4—O6	120.31 (10)	C5—C6—H6A	109.5
O8—B5—O4	122.14 (10)	C5—C6—H6B	109.5
O8—B5—O6	116.87 (10)	H6A—C6—H6B	109.5
O4—B5—O6	120.99 (10)	C5—C6—H6C	109.5
C1—N2—C3	108.62 (10)	H6A—C6—H6C	109.5
C1—N2—C4	124.99 (11)	H6B—C6—H6C	109.5
C3—N2—C4	126.39 (11)		

### Hydrogen-bond geometry (Å, º)

**Table:**

*D*—H···*A*	*D*—H	H···*A*	*D*···*A*	*D*—H···*A*
O7—H1···O9^i^	0.88 (2)	1.84 (2)	2.7169 (12)	172.8 (17)
O8—H2···O2^ii^	0.861 (18)	1.843 (18)	2.7022 (12)	174.9 (17)
O9—H3···O3^iii^	0.87 (2)	1.80 (2)	2.6687 (11)	174.3 (18)
O10—H4···O4^iv^	0.85 (2)	1.90 (2)	2.7426 (12)	173.9 (19)
C1—H1*A*···O5^iii^	0.95	2.14	3.0265 (14)	155
C4—H4*C*···O10^ii^	0.98	2.47	3.4456 (16)	175

Symmetry codes: (i) *x*+1, *y*, *z*; (ii) *x*, −*y*+1/2, *z*−1/2; (iii) −*x*+2, −*y*, −*z*+1; (iv) *x*, −*y*+1/2, *z*+1/2.
